# 9-Ethyl-9*H*-carbazole-3-carbaldehyde

**DOI:** 10.1107/S1600536810025183

**Published:** 2010-07-03

**Authors:** Mao-Sen Yuan, Li Zhao, Ran-rong Zhang

**Affiliations:** a College of Science, Northwest A&F University, Yangling 712100, Shannxi Province, People’s Republic of China; b Hospital of Northwest A&F University, Yangling 712100, Shannxi Province, People’s Republic of China

## Abstract

The title mol­ecule, C_15_H_13_NO, approximates a planar conformation except for the alkyl chain (ethyl group) bonded to the N atom with a maximum deviation from the least-squares plane through the 15 planar atoms of 0.120 (2) Å for the O atom. The distance of the formyl O atom from the plane of the carbazole ring is 0.227 (2) Å. The N—C bond lengths in the central ring are significantly different, reflecting the electron-withdrawing properties of the aldehyde group. As a consequence, charge transfer may occur from the carbazole N atom to the substituted benzene ring.

## Related literature

For the properties of carbazole derivatives, see: van Dijken *et al.* (2004 ▶); Li *et al.* (2005 ▶). For the X-ray structure of 9-ethyl-3,6-diformyl-9*H*-carbazole, see: Wang *et al.* (2008 ▶) and of 9-ethyl-9*H*-carbazole, see: Kimura *et al.* (1985 ▶).
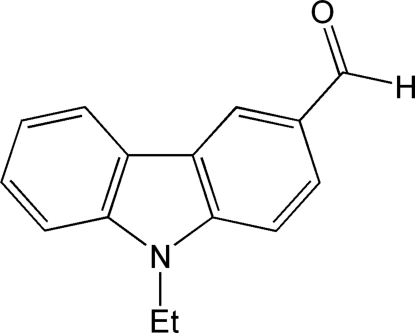


## Experimental

### 

#### Crystal data

C_15_H_13_NO
*M* *_r_* = 223.26
Monoclinic, 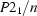

*a* = 10.6523 (10) Å
*b* = 8.2312 (6) Å
*c* = 13.8005 (12) Å
β = 104.387 (1)°
*V* = 1172.10 (17) Å^3^
*Z* = 4
Mo *K*α radiation
μ = 0.08 mm^−1^
*T* = 298 K
0.50 × 0.44 × 0.43 mm

#### Data collection

Bruker APEXII CCD area-detector diffractometer
Absorption correction: multi-scan (*SADABS*; Bruker, 2005 ▶) *T*_min_ = 0.961, *T*_max_ = 0.967
5763 measured reflections
2065 independent reflections
1313 reflections with *I* > 2σ(*I*)
*R*_int_ = 0.029

#### Refinement

*R*[*F* ^2^ > 2σ(*F* ^2^)] = 0.044
*wR*(*F* ^2^) = 0.116
*S* = 1.05
2065 reflections
155 parameters
H-atom parameters constrained
Δρ_max_ = 0.13 e Å^−3^
Δρ_min_ = −0.11 e Å^−3^

### 

Data collection: *APEX2* (Bruker, 2005 ▶); cell refinement: *SAINT* (Bruker, 2005 ▶); data reduction: *SAINT*; program(s) used to solve structure: *SHELXS97* (Sheldrick, 2008 ▶); program(s) used to refine structure: *SHELXL97* (Sheldrick, 2008 ▶); molecular graphics: *SHELXTL* (Sheldrick, 2008 ▶); software used to prepare material for publication: *SHELXTL*.

## Supplementary Material

Crystal structure: contains datablocks global, I. DOI: 10.1107/S1600536810025183/bh2297sup1.cif

Structure factors: contains datablocks I. DOI: 10.1107/S1600536810025183/bh2297Isup2.hkl

Additional supplementary materials:  crystallographic information; 3D view; checkCIF report

## Figures and Tables

**Fig. 1.**
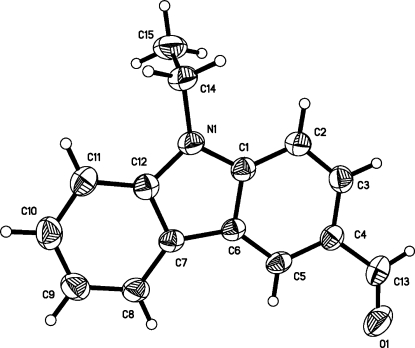
The molecular structure of (I). Displacement ellipsoids are drawn at the 30% probability level.

**Fig. 2.**
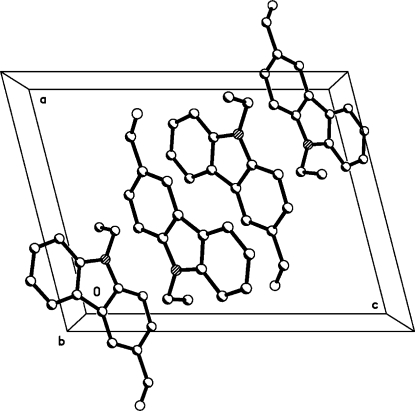
A view of the molecular packing of (I) along axis b.

## supplementary crystallographic information

### Comment

Carbazole is a conjugated unit which has interesting optical and electronic properties. A number of carbazole derivatives have been designed and synthesized to be used as luminescent materials and hole-transporting materials (van Dijken *et al.*, 2004; Li *et al.*, 2005). In the course of exploring new luminescent compounds, we obtained an intermediate compound, 9-ethyl-3-formyl-9*H*-carbazole (I). Here we report the structure and synthesis of (I).

The molecule (Fig. 1) lies approximately in a plane besides the alkyl chain (ethyl group). There is a minor displacement between the oxygen atom O1 and the plane of the carbazole ring. And the distance from the oxygen atom O1 to the carbazole plane (the least-squares plane defined by all the 13 atoms of the carbazole framework) is 0.227 (2) Å. The remarkable difference of N—C bond lengths is observed in this structure: N1—C1 = 1.372 (3), N1—C12 = 1.391 (3) Å, which is obviously different from that of 9-ethyl-3,6-diformyl-9*H*-carbazole (Wang *et al.*, 2008) and that of 9-ethyl-9*H*-carbazole (Kimura *et al.*, 1985). The different N—C bond lengths maybe root from the structural asymmetry. The pull-electron property of aldehyde group induces a charge-transfer from nitrogen atom N1 to the benzene ring which connects with the aldehyde group.

The molecules are packed in *P*2_1_/*n* space group, which is the same as for 9-ethyl-3,6-diformyl-9*H*-carbazole, but different from that of 9-ethyl-9*H*-carbazole (*Pbca*). There are no classic hydrogen bonds in this structure. However, the weak intermolecular interaction C11—H11···O1 (symmetry code for O1: *x*-1, *y*, *z*), is helpful to the stabilization of the crystal structure (Fig. 2). This intermolecular hydrogen bond is characterized by the H11···O1 separation of 2.54 Å.

### Experimental

9-Ethyl-9*H*-carbazole (0.30 g, 1.54 mmol) was dissolved in *N*,*N*-dimethylformamide (DMF, 10 ml). After cooling the mixture to 273 K, a DMF solution of POCl_3_ (0.24 g, 1.60 mmol) was slowly added. After stirring for 10 h., the mixture was poured into ice water and further stirred for 0.5 h. The solution was extracted with chloroform and dried over Na_2_SO_4_. After removing the solvent, the crude product was purified by recrystallization from ethanol, affording the title compound, (I) (0.29 g, 85%). Then, compound (I) was dissolved in a mixture of solvents, chloroform and hexane, and colorless block crystals were formed on slow evaporation at room temperature over one week.

### Refinement

All H atoms were placed in geometrically calculated positions and refined using a riding model with C—H = 0.93 Å (for aromatic CH), C—H = 0.97 %A (for CH_2_ groups), and 0.96 %A (for CH_3_ groups). Their isotropic displacement parameters were set to 1.2 times (1.5 times for CH_3_ groups) the equivalent displacement parameter of their parent atoms.

### Crystal data

**Table:**

C_15_H_13_NO	*F*(000) = 472
*M**_r_* = 223.26	*D*_x_ = 1.265 Mg m^−^^3^
Monoclinic, *P*2_1_/*n*	Mo *K*α radiation, λ = 0.71073 Å
Hall symbol: -P 2yn	Cell parameters from 1960 reflections
*a* = 10.6523 (10) Å	θ = 2.8–25.2°
*b* = 8.2312 (6) Å	µ = 0.08 mm^−^^1^
*c* = 13.8005 (12) Å	*T* = 298 K
β = 104.387 (1)°	Block, colorless
*V* = 1172.10 (17) Å^3^	0.50 × 0.44 × 0.43 mm
*Z* = 4	

### Data collection

**Table:**

Bruker APEXII CCD area-detector diffractometer	2065 independent reflections
Radiation source: fine-focus sealed tube	1313 reflections with *I* > 2σ(*I*)
graphite	*R*_int_ = 0.029
φ and ω scans	θ_max_ = 25.0°, θ_min_ = 2.2°
Absorption correction: multi-scan (*SADABS*; Bruker, 2005)	*h* = −12→12
*T*_min_ = 0.961, *T*_max_ = 0.967	*k* = −9→5
5763 measured reflections	*l* = −16→16

### Refinement

**Table:**

Refinement on *F*^2^	Primary atom site location: structure-invariant direct methods
Least-squares matrix: full	Secondary atom site location: difference Fourier map
*R*[*F*^2^ > 2σ(*F*^2^)] = 0.044	Hydrogen site location: inferred from neighbouring sites
*wR*(*F*^2^) = 0.116	H-atom parameters constrained
*S* = 1.05	*w* = 1/[σ^2^(*F*_o_^2^) + (0.0369*P*)^2^ + 0.3721*P*] where *P* = (*F*_o_^2^ + 2*F*_c_^2^)/3
2065 reflections	(Δ/σ)_max_ < 0.001
155 parameters	Δρ_max_ = 0.13 e Å^−^^3^
0 restraints	Δρ_min_ = −0.11 e Å^−^^3^
0 constraints	

### Fractional atomic coordinates and isotropic or equivalent isotropic displacement parameters (Å^2^)

**Table:**

	*x*	*y*	*z*	*U*_iso_*/*U*_eq_	
N1	0.23808 (16)	0.0604 (2)	0.36043 (12)	0.0579 (5)	
O1	0.83067 (17)	0.1354 (3)	0.36513 (14)	0.0966 (6)	
C1	0.3591 (2)	0.0376 (3)	0.34470 (15)	0.0518 (5)	
C2	0.3972 (2)	−0.0683 (3)	0.27949 (16)	0.0631 (6)	
H2	0.3382	−0.1383	0.2390	0.076*	
C3	0.5250 (2)	−0.0665 (3)	0.27648 (16)	0.0626 (6)	
H3	0.5528	−0.1382	0.2340	0.075*	
C4	0.6142 (2)	0.0397 (3)	0.33530 (15)	0.0545 (6)	
C5	0.5753 (2)	0.1450 (3)	0.40056 (14)	0.0529 (5)	
H5	0.6347	0.2157	0.4401	0.063*	
C6	0.44792 (19)	0.1444 (2)	0.40663 (13)	0.0466 (5)	
C7	0.37450 (19)	0.2342 (2)	0.46404 (14)	0.0489 (5)	
C8	0.4063 (2)	0.3526 (3)	0.53694 (16)	0.0639 (6)	
H8	0.4914	0.3886	0.5592	0.077*	
C9	0.3104 (3)	0.4162 (3)	0.57591 (18)	0.0766 (7)	
H9	0.3304	0.4973	0.6242	0.092*	
C10	0.1842 (3)	0.3611 (3)	0.54428 (19)	0.0773 (7)	
H10	0.1211	0.4063	0.5720	0.093*	
C11	0.1490 (2)	0.2417 (3)	0.47318 (17)	0.0666 (7)	
H11	0.0640	0.2049	0.4524	0.080*	
C12	0.2467 (2)	0.1786 (3)	0.43386 (15)	0.0531 (5)	
C13	0.7479 (2)	0.0386 (3)	0.32700 (18)	0.0709 (7)	
H13	0.7716	−0.0439	0.2890	0.085*	
C14	0.1223 (2)	−0.0304 (3)	0.31232 (18)	0.0747 (7)	
H14A	0.1251	−0.0561	0.2443	0.090*	
H14B	0.0467	0.0368	0.3092	0.090*	
C15	0.1098 (3)	−0.1842 (4)	0.3666 (2)	0.1012 (10)	
H15A	0.1832	−0.2527	0.3679	0.152*	
H15B	0.0318	−0.2395	0.3329	0.152*	
H15C	0.1065	−0.1593	0.4339	0.152*	

### Atomic displacement parameters (Å^2^)

**Table:**

	*U*^11^	*U*^22^	*U*^33^	*U*^12^	*U*^13^	*U*^23^
N1	0.0484 (11)	0.0677 (12)	0.0556 (11)	−0.0109 (9)	0.0089 (8)	−0.0058 (10)
O1	0.0640 (12)	0.1300 (18)	0.1015 (14)	−0.0201 (12)	0.0310 (10)	0.0030 (13)
C1	0.0529 (13)	0.0550 (13)	0.0474 (12)	−0.0049 (10)	0.0122 (10)	0.0044 (10)
C2	0.0631 (15)	0.0672 (16)	0.0590 (14)	−0.0122 (12)	0.0152 (11)	−0.0083 (12)
C3	0.0731 (17)	0.0609 (15)	0.0577 (14)	0.0011 (12)	0.0239 (12)	−0.0005 (12)
C4	0.0584 (14)	0.0573 (14)	0.0494 (12)	−0.0012 (11)	0.0169 (10)	0.0115 (11)
C5	0.0529 (13)	0.0560 (13)	0.0480 (12)	−0.0121 (10)	0.0093 (10)	0.0098 (11)
C6	0.0495 (12)	0.0483 (12)	0.0411 (11)	−0.0056 (10)	0.0093 (9)	0.0083 (10)
C7	0.0567 (13)	0.0473 (12)	0.0427 (11)	−0.0095 (10)	0.0123 (9)	0.0086 (10)
C8	0.0803 (17)	0.0608 (15)	0.0553 (13)	−0.0202 (13)	0.0257 (12)	−0.0002 (12)
C9	0.109 (2)	0.0605 (16)	0.0702 (16)	−0.0180 (15)	0.0410 (15)	−0.0074 (13)
C10	0.095 (2)	0.0729 (17)	0.0764 (17)	0.0029 (16)	0.0449 (15)	0.0022 (15)
C11	0.0623 (15)	0.0761 (17)	0.0652 (14)	−0.0018 (13)	0.0228 (12)	0.0092 (14)
C12	0.0581 (14)	0.0559 (13)	0.0454 (11)	−0.0020 (11)	0.0129 (10)	0.0079 (11)
C13	0.0654 (17)	0.0861 (19)	0.0677 (16)	0.0026 (14)	0.0289 (13)	0.0157 (14)
C14	0.0548 (15)	0.097 (2)	0.0704 (15)	−0.0197 (13)	0.0114 (12)	−0.0166 (15)
C15	0.108 (2)	0.108 (2)	0.092 (2)	−0.0571 (19)	0.0328 (17)	−0.0225 (19)

### Geometric parameters (Å, °)

**Table:**

N1—C1	1.372 (3)	C7—C12	1.398 (3)
N1—C12	1.391 (3)	C8—C9	1.371 (3)
N1—C14	1.453 (3)	C8—H8	0.9300
O1—C13	1.208 (3)	C9—C10	1.382 (3)
C1—C2	1.384 (3)	C9—H9	0.9300
C1—C6	1.413 (3)	C10—C11	1.374 (3)
C2—C3	1.372 (3)	C10—H10	0.9300
C2—H2	0.9300	C11—C12	1.388 (3)
C3—C4	1.394 (3)	C11—H11	0.9300
C3—H3	0.9300	C13—H13	0.9300
C4—C5	1.385 (3)	C14—C15	1.494 (4)
C4—C13	1.457 (3)	C14—H14A	0.9700
C5—C6	1.380 (3)	C14—H14B	0.9700
C5—H5	0.9300	C15—H15A	0.9600
C6—C7	1.447 (3)	C15—H15B	0.9600
C7—C8	1.381 (3)	C15—H15C	0.9600
			
C1—N1—C12	108.45 (16)	C8—C9—C10	120.8 (2)
C1—N1—C14	125.50 (19)	C8—C9—H9	119.6
C12—N1—C14	125.96 (19)	C10—C9—H9	119.6
N1—C1—C2	128.9 (2)	C11—C10—C9	122.1 (2)
N1—C1—C6	109.47 (18)	C11—C10—H10	118.9
C2—C1—C6	121.60 (19)	C9—C10—H10	118.9
C3—C2—C1	117.8 (2)	C10—C11—C12	116.7 (2)
C3—C2—H2	121.1	C10—C11—H11	121.7
C1—C2—H2	121.1	C12—C11—H11	121.7
C2—C3—C4	121.8 (2)	C11—C12—N1	128.8 (2)
C2—C3—H3	119.1	C11—C12—C7	122.0 (2)
C4—C3—H3	119.1	N1—C12—C7	109.22 (18)
C5—C4—C3	120.0 (2)	O1—C13—C4	125.7 (3)
C5—C4—C13	120.7 (2)	O1—C13—H13	117.1
C3—C4—C13	119.2 (2)	C4—C13—H13	117.1
C6—C5—C4	119.61 (19)	N1—C14—C15	112.2 (2)
C6—C5—H5	120.2	N1—C14—H14A	109.2
C4—C5—H5	120.2	C15—C14—H14A	109.2
C5—C6—C1	119.12 (19)	N1—C14—H14B	109.2
C5—C6—C7	134.79 (19)	C15—C14—H14B	109.2
C1—C6—C7	106.09 (17)	H14A—C14—H14B	107.9
C8—C7—C12	119.5 (2)	C14—C15—H15A	109.5
C8—C7—C6	133.74 (19)	C14—C15—H15B	109.5
C12—C7—C6	106.76 (18)	H15A—C15—H15B	109.5
C9—C8—C7	118.9 (2)	C14—C15—H15C	109.5
C9—C8—H8	120.6	H15A—C15—H15C	109.5
C7—C8—H8	120.6	H15B—C15—H15C	109.5
			
C12—N1—C1—C2	179.4 (2)	C1—C6—C7—C12	−0.3 (2)
C14—N1—C1—C2	2.6 (3)	C12—C7—C8—C9	−1.9 (3)
C12—N1—C1—C6	−1.4 (2)	C6—C7—C8—C9	178.8 (2)
C14—N1—C1—C6	−178.23 (19)	C7—C8—C9—C10	1.1 (3)
N1—C1—C2—C3	179.0 (2)	C8—C9—C10—C11	0.0 (4)
C6—C1—C2—C3	−0.1 (3)	C9—C10—C11—C12	−0.2 (3)
C1—C2—C3—C4	−1.2 (3)	C10—C11—C12—N1	−178.6 (2)
C2—C3—C4—C5	1.4 (3)	C10—C11—C12—C7	−0.6 (3)
C2—C3—C4—C13	−178.5 (2)	C1—N1—C12—C11	179.4 (2)
C3—C4—C5—C6	−0.2 (3)	C14—N1—C12—C11	−3.8 (3)
C13—C4—C5—C6	179.62 (18)	C1—N1—C12—C7	1.2 (2)
C4—C5—C6—C1	−1.0 (3)	C14—N1—C12—C7	178.0 (2)
C4—C5—C6—C7	−179.8 (2)	C8—C7—C12—C11	1.7 (3)
N1—C1—C6—C5	−178.10 (17)	C6—C7—C12—C11	−178.88 (19)
C2—C1—C6—C5	1.2 (3)	C8—C7—C12—N1	−179.94 (17)
N1—C1—C6—C7	1.1 (2)	C6—C7—C12—N1	−0.5 (2)
C2—C1—C6—C7	−179.68 (19)	C5—C4—C13—O1	−8.4 (3)
C5—C6—C7—C8	−2.0 (4)	C3—C4—C13—O1	171.5 (2)
C1—C6—C7—C8	179.0 (2)	C1—N1—C14—C15	86.1 (3)
C5—C6—C7—C12	178.6 (2)	C12—N1—C14—C15	−90.2 (3)
